# Protein Interaction Analysis and Molecular Simulation of the Anti-Inflammatory Activities in *Melaleuca cajuputi* Extract Against COVID‐19

**DOI:** 10.1155/ijin/5568294

**Published:** 2024-11-28

**Authors:** Agustyas Tjiptaningrum, Intanri Kurniati, Fadilah Fadilah, Tiwuk Susantiningsih, Aisyah Fitriannisa Prawiningrum, Wahyu Dian Utari, Linda Erlina

**Affiliations:** ^1^Doctoral Program of Biomedical Science, Faculty of Medicine, University of Indonesia, Jakarta, Indonesia; ^2^Department of Clinical Pathology of University of Lampung, Bandar Lampung, Indonesia; ^3^Department of Medical Chemistry, Faculty of Medicine, Universitas Indonesia, Jakarta, Indonesia; ^4^Bioinformatics Core Facilities, Indonesian Medical Education and Research Institute (IMERI), Faculty of Medicine, Universitas Indonesia, Jakarta, Indonesia

## Abstract

Coronavirus disease-19 (COVID-19) is correlated to a severe condition caused by a cytokine storm during which numerous proinflammatory cytokines, including interleukin-6 (IL-6) are released. IL-6 is a critical driver in the COVID-19 inflammatory state, and the inhibition is considered a potential treatment approach to prevent serious complications. Meanwhile, *Melaleuca cajuputi* is a plant with antibacterial, antiviral, anti-inflammatory, and antioxidant activities. Therefore, this aimed to investigate the anti-inflammatory potential of *M. cajuputi* in silico. Extraction of leaves was conducted by using 96% ethanol, followed by fractionation to obtain active compounds. Subsequently, LC/MS and GC/MS analyses were performed to obtain active compound profiling. Protein-protein interaction (PPI), as well as molecular docking and dynamic analyses, were performed to examine interaction of active compounds of *M. cajuputi* with IL-6. The results showed that 30 protein nodes played a significant role in COVID-19 cytokine storm and eight active compounds had interactions with IL-6. Among the active compounds, pinostrobin chalcone had the best delta G interaction with IL-6. In conclusion, *M. cajuputi* has potential activity as an anti-inflammatory agent against COVID-19.

## 1. Introduction

Severe acute respiratory syndrome coronavirus-2 (SARS-CoV-2), the coronavirus disease-19 (COVID-19) virus was discovered in Wuhan, China, toward the end of 2019. Subsequently, the virus has transmitted quickly worldwide, becoming a significant public health problem. As stated in various reports, COVID-19, which primarily causes pneumonia, poses a threat to global health [1, 2]. In this context, a common complication often observed is cytokine storms, a condition where superactivation of the immune system leads to uncontrollable cytokine release in lung tissues. The term “cytokine storm” refers to a series of events that cause multiple organ failures and death, with the serious clinical complication being acute respiratory distress syndrome (ARDS) [3, 4].

Based on the evidence, proinflammatory cytokines have a control in the COVID-19 pathogenesis and complications [5–7]. Therefore, managing cytokine release syndrome (CRS) and preventing subsequent infections may be a valuable approach. This underscores the urgent need for anti-inflammatory drugs to treat COVID-19 patients with CRS. The identification of the underlying mechanisms can help develop treatment strategies and accelerate recovery. Several clinical trials are currently underway to investigate new supportive care and interventions to treat this infection [8, 9].

The host immune system can induce a deadly inflammatory situation known as CRS in COVID-19 patients. CRS occurs when the virus enters and replicates alveolar epithelial cells, and then the virions are released and captured by phagocytic cells including dendritic cells, macrophages, or neutrophils. The damage of epithelial cells, virion infection, and a phagocytic-captured process leads to the massive release of proinflammatory cytokine. This release increases the systemic level of IL-6, IL-1b, TNF, and other proinflammatory cytokines, subsequently causing cytokine storm and a severe condition of COVID-19 [3, 10].

The recent analysis reported excessive certain cytokines production, including IL-6, can develop into the primary cause of inflammatory response. Therefore, anti-inflammatory therapy may be a promising intervention for COVID-19–related pneumonia [3, 11].

*Melaleuca cajuputi* is among hundreds of *Melaleuca* species (Myrtaceae family) grown in Indonesia, Vietnam, and Thailand [12]. The essential oil, in particular, is widely used for medicinal purposes to treat coughs and colds, as a general muscle relaxant, laxative, and sedative. It reportedly has antifungal, anti-inflammatory, and antioxidant properties [13–15]. According to some traditional remedies, cajeput oil is used to inhibit certain bacteria and viruses. Given the development of current data both related to the SARS-CoV-2 target pathway and anti-SARS-CoV-2 candidate inhibitor compounds, it is necessary to find a drug for these purposes using molecular modeling approaches. The approaches include protein-protein interaction (PPI), protein-drug interaction, molecular protein docking interaction test, and molecular dynamics to determine drug interactions in target. Therefore, this analysis examined the *M. cajuputi*'s anti-inflammatory potential by docking the active compounds found in the plant with IL-6.

## 2. Materials and Methods

### 2.1. Extraction of *M. cajuputi*

*M. cajuputi* leaves used for the study were harvested in Indonesia, and then using the Soxhlet apparatus, 100 g of the powder was converted into aqueous, methanol, ethanol, and ethyl acetate hot crude extracts. Excess solvents were removed by concentrating and storing the extract in desiccators. For comparison of the anti-inflammatory activity of the extract, the dexamethasone was used as the existing standard anti-inflammatory drug for COVID-19.

### 2.2. Active Compounds Profiling Using GC-MS

The Agilent-7890 A GC instrument was used for the GC-MS analysis, along with an MS-5975 inert MSD and a triple-axis mass selective ion detector. In addition, a DB-5MS column of 30 × 0.2 mm capillary column was applied. The starting temperature was maintained at 150°C, with a 300°C maximum temperature. Using split mode, 1L of the sample was injected (10:1) and helium gas was applied as a carrier at a 0.8 mL/min flow rate for 22 min. For GC-MS identification of phytocomponents, the National Institute of Standards and Technology MS library (NIST-MS library) database was used.

### 2.3. Construction of a Database for Host Target Proteins Associated With Inflammation Process Related to COVID-19

The target proteins were retrieved from GeneCard available at https://www.genecards.org [16]. Using the first keyword “SARS CoV 2” in the search engine column, a total of 5306 genes were retrieved. The second keyword “inflammation” was later added by using [AND], yielding 3653 genes. The third keyword “cytokine storm” was imputed to the search engine column and the genes hit 496. The fourth keyword “ARDS” was imputed and the genes hit 306. All data were then downloaded in .csv format (306 genes) with gene symbol, gene description name, category, GIFtS, GC id, and score.

#### 2.3.1. Prediction of Significance Target Proteins

PPI was created using Cytoscape version 3.9.1 (https://cytoscape.org) [17]. Then, data were uploaded in .csv format as unassigned tables. The category of proteins was filtered to include protein-coding only resulting in a total of 301, while pseudogenes and RNA-gene were excluded. PPI was performed by using the STRING database [18] integrated with Cytoscape 3.9.1. The parameter of Stringdb total score was set at the highest confidence (0.990–0.999), and the rank of significance protein was based on the degree of connectivity score. Hub genes analysis was added to decide the centrality of the protein interactions with parameter maximal clique centrality (MCC), maximum neighborhood component (MNC), and betweenness using molecular complex detection (MCODE), CytoHubba plugin. The parameter for filtration was set: MCODE scores > 10, degree cut-off = 2, node score cut-off = 0.2, Max. Depth = 100, and *k*-score = 2 [19].

#### 2.3.2. Gene Ontology (GO) and Kyoto Encyclopedia of Genes and Genomes (KEGG) Pathway Analyses

GO and KEGG analyses of the candidate genes were carried out applying Stringdb online tool (https://string-db.org) and Enrichr (https://maayanlab.cloud/Enrichr/) [20]. The enrichment significance threshold was arranged to *p* < 0.05, while the GO analysis covered molecular function, biological process, cellular component, and human phenotype ontology. The q-value (adjusted *p* value) obtained applying the Benjamini–Hochberg method for correcting multiple hypotheses and was also considered significant when < 0.05. In addition, Shiny GO version 0.76.3 (https://bioinformatics.sdstate.edu/go/) was used for constructing the KEGG pathway related to significant protein [21].

### 2.4. Drug-Likeness of Compounds From *M. cajuputi* Extract

Before molecular docking, compounds found in *M. Cajuputi* extract were filtered based on the drug-likeness and other properties such as distribution, toxicity, absorption, and metabolism which were estimated by applying SwissADME (https://swissadme.ch/) [22] and ProTox II (https://tox-new.charite.de/protox_II) [23]. This study analyzed the interaction between activated compounds of the ethanol extract of *M. cajuputi* leaves and human IL-6 (PDB ID: 1ALU). Therefore, QSAR analysis was not conducted.

### 2.5. Molecular Docking

Human IL-6 structure was retrieved from https://www.rcsb.org.in.pdb format (PDB ID: 1ALU) [24]. It was chosen because it has a high resolution of 1.90 Å and absence of mutation. The chain receptor was separated from the cocrystalized ligand. The protein preparation process included hydrogenating, computing charges, and removing water. Active compounds found in plant extract, available in .sdf format, were obtained from the PubChem database and a cocrystalized ligand, (+) -tartaric acid, applied as a positive control. The docking procedures were prepared using the AutoDockTools software (version 1.5.6, at https://autodock.scripps.edu), a GUI program (graphical user interface) for configuring [25]. The grid box volume was set to 60 × 60 × 60 with 0.375 spacing, and total docking runs was arranged to 10. In addition, the grid box position was placed on the cocrystalized ligand center to serve as a binding site.

### 2.6. Molecular Dynamic Simulation

The .dlg file output from molecular docking was saved in .pdb complex to serve as a raw file for the molecular dynamic simulation using YASARA version 22.9.24 (licensed to fadilah.msi@ui.ac.id). The force field parameters were set using AMBER 14 with timesteps of 10,000, save interval of 10,000, water mode set to cube, and duration of 100 ns [26].

## 3. Results

### 3.1. Active Compounds Profiling

Table 1 shows the chemical composition of the liquid extract of *M. cajuputi* leaves obtained from mass spectrometry GC/MS examination.

### 3.2. Protein-Protein Interaction

From the construction of PPI using Cytoscape 3.9.1 (protein-coding only) and Stringdb database, the total node of protein was 301 with 6440 edges. The Stringdb score ranged between 0.400 and 0.999, which was calculated using experiments, databases, gene fusion, text mining, gene neighborhood, coexpression, and interspecies. The highest interaction confidence was observed with scores between 0.900 and 0.999. By filtering for a confidence Stringdb database score of 0.999, the total nodes were refined to 88 with 1619 edges. For increased specificity, the experiment parameter was set to 0.967–0.999, resulting in a network of 30 nodes and 164 edges.

All the nodes (proteins) were extracted by the protein symbol and submitted to Enrichr and String-db web server to develop the functional enrichments including GO, DEG, and KEGG pathway analyses.

**Figure 1:**
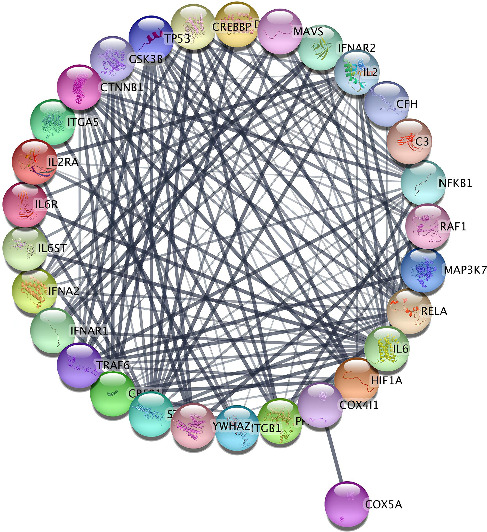
PPI of 30 nodes and 164 edges.

Figure 1 shows the result of PPI. Based on the PPI results from String-db, the total number of nodes was 30, with 164 edges. The PPI enrichment *p* value < 1.0e − 16, the average node degree was 10.9, and the average local clustering coefficient was 0.769 (Figure 1).

Table 2 shows the CytoHubba results, seven genes (NFKB1, IL6, TP53, STAT1, HIF1A, RELA, and CTNNB1) as hub genes were identified by the CytoHubba plugin using top 10 of MCC, MNC, and betweenness score.

**Figure 2:**
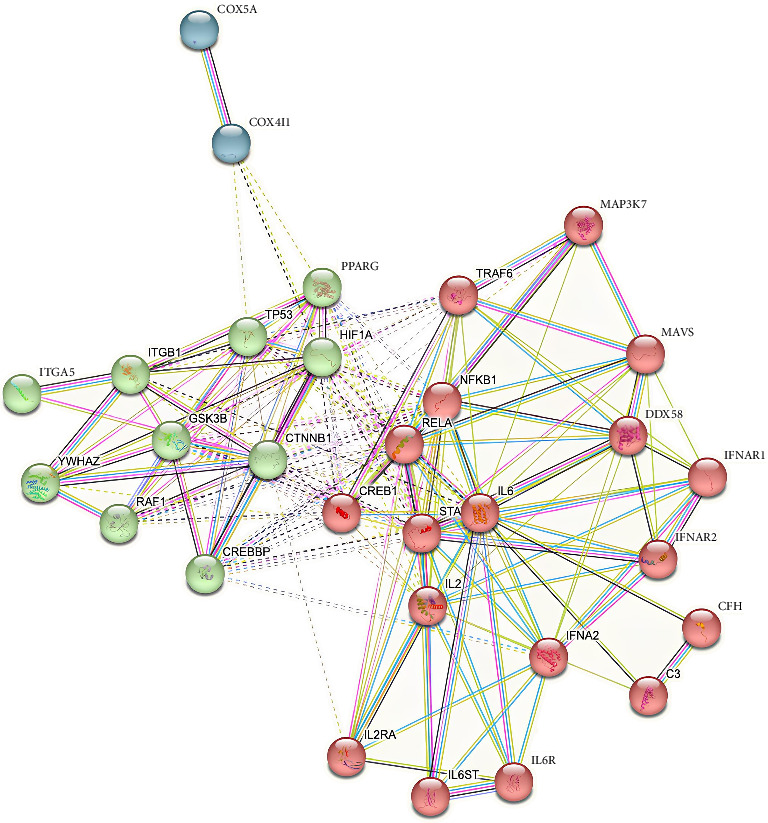
K-means clustering of significant PPI (Cluster 1-red, Cluster 2-green, and Cluster 3-blue) (stringDb).

Based on the 30 significant protein nodes, *K*-means clustering was used to categorize protein into three clusters. Specifically, Cluster 1, represented in red nodes, included 18 proteins, Cluster 2 in green nodes had 10 proteins, and Cluster 3 in blue nodes had two proteins (Figure 2). IL-6 biomarker had 17 interactions with other proteins; hence, this protein was selected to continue for molecular docking and dynamic simulation process.

### 3.3. GO Analysis

The GO analysis was performed using String-db, Enrichr, and ShinyGO with functional enrichment of molecular function, pathway, cellular component, biological process, and human phenotype ontology.

**Figure 3:**
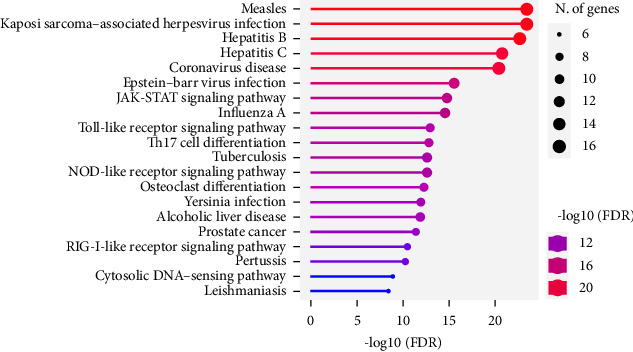
Predicted network analysis.

Figure 3 shows the GO analysis result. The top 20 predicted pathways were compiled into a network and the coronavirus disease included in the top 5. Based on the results, the number of enrichment FDR values was 3.6E − 21.

**Figure 4:**
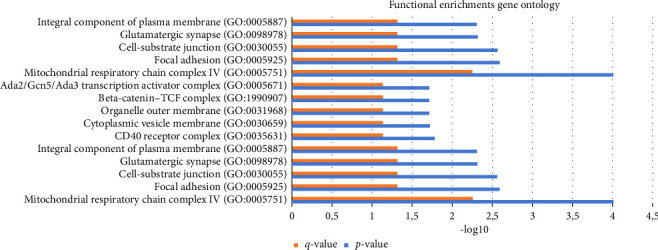
Functional enrichments GO (biological process, molecular function, and cellular components).

Based on the analysis results shown in the bar chart (Figure 4), the top five biological processes include regulation of cellular response on cytokine stimulus, cytokine-mediated signaling pathway, cytokine production's positive regulation, apoptotic process's negative regulation, and regulation of apoptotic process. The top five molecular functions include RNA polymerase II–specific DNA-binding transcription factor, DNA-binding transcription factor, ubiquitin-like protein ligase, kinase, and protein-kinase–binding. Meanwhile, the top five cellular components include mitochondrial respiratory chain complex IV, focal adhesion, cell-substrate junction, glutamatergic synapse, and integral component of plasma membrane.

**Figure 5:**
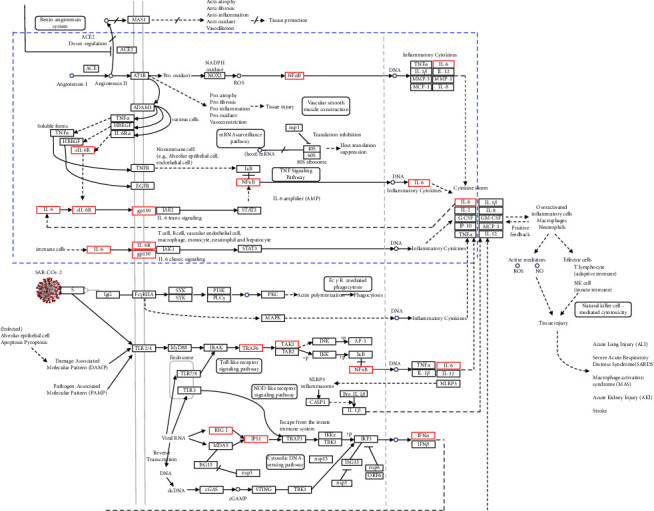
Pathway of coronavirus disease (COVID-19) from KEGG.

Figure 5 shows the mechanism of the COVID-19 cytokine storm from KEGG, with the blue box indicating proteins identified in the PPI result. Proteins within the dotted lines are potential targets for the anti-inflammatory effects of the ethanol extract from *M. cajuputi* leaves.

Based on the COVID-19 pathway (Figure 5), the proposed mechanism of action of the ethanol extract from *M. cajuputi* leaves as an anti-inflammatory agent is the inhibition of NF-*κ*B expression as a transcription factor for proinflammatory cytokines. This inhibition will cause a decrease in the expression of proinflammatory proteins. Another mechanism is the inhibition of the activity of proinflammatory cytokines. This inhibition causes a decrease in inflammatory activity, reducing the possibility of cytokine storm condition.

### 3.4. Drug-Likeness and ADMETox Prediction

Based on drug-likeness and ADMETox prediction using SwissADME and ProTox II, all active compounds were mostly nontoxic with toxicity class between 4 and 5. The compounds met the drug-likeness criteria including acceptable hydrogen bond donor, and acceptor, logP, as well as molecular weight. In addition, these compounds are noninhibitors of CYP P450 (Table S1: drug-likeness and ADMETOX prediction).

### 3.5. Molecular Docking

Table 3 shows the molecular docking between active compounds of the ethanol extract of *M. cajuputi* leaves and IL-6. The study used L (+) -tartaric acid as a native ligand. Among the 10 compounds from the liquid ethanol extract of *M. cajuputi* leaves, pinostrobin chalcone had the lowest binding affinity −6.08 kcal/mol and inhibition constant 34.77 μM, followed by L (+) -tartaric acid (native ligand) with −5.40 kcal/mol binding energy and 110.71 μM inhibition constant.

**Figure 6:**
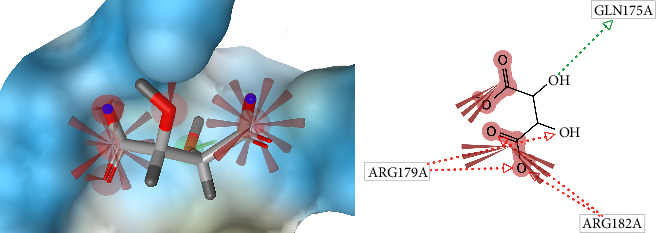
Native ligand (L (+)–tartaric acid) conformation in the binding site of IL-6 (3D visualization–A and 2D visualization–B).

Figure 6 is a visualization of native ligand (L (+) -tartaric acid) conformation in the active binding site of IL-6. The native ligand makes an interaction and binds to the active site of IL-6. This picture was generated by LigandScout 4.3 software.

**Figure 7:**
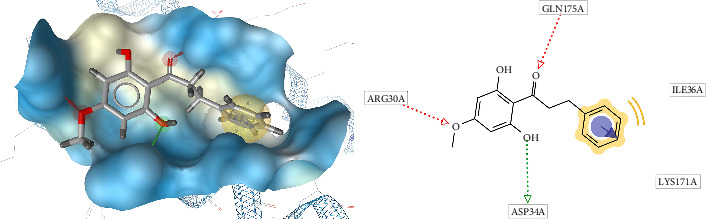
Pinostrobin chalcone conformation in the binding site of IL-6 (3D visualization–A and 2D visualization–B).

Figure 7 shows the conformation of pinostrobin chalcone in the binding site of ILs-6. Molecular docking posing for native ligand (L (+) -tartaric acid) with IL-6 receptor binding site shows several important amino acid residues such as Gln175, Arg179, and Arg182. Through comparison with pinostrobin chalcone conformation in the binding site of IL-6, Gln175, Ile36, Lys171, Asp34, and Arg30 were found to play an important role in enhancing ligand affinity. Pinostrobin chalcone showed the best interaction with the IL-6 active site, namely, Gln175, Ile36, Lys171, Asp34, and Arg30 due to the low binding affinity and inhibition constant.

**Figure 8:**
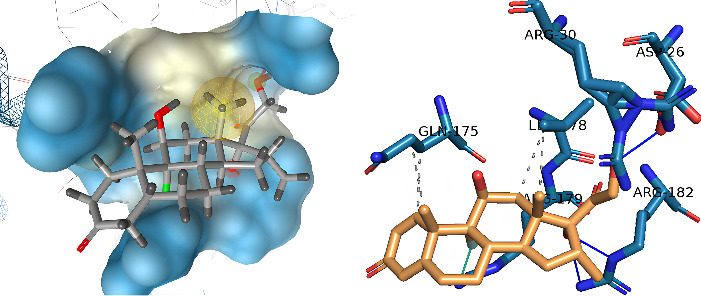
Molecular docking of IL-6 and dexamethasone.

Figure 8 shows the result of molecular docking of IL-6 with dexamethasone. The binding affinity score is −5.27 kcal/mol with several interactions such as hydrophobic interaction Gln175 and Leu178, and hydrogen bonds Asp26, Arg30, and Arg182. The binding affinity of dexamethasone–IL-6 is higher than the binding affinity of pinostrobin chalcone-IL-6. It showed that the interaction between pinostrobin chalcone with IL-6 was better than the interaction between dexamethasone–L-6.

**Figure 9:**
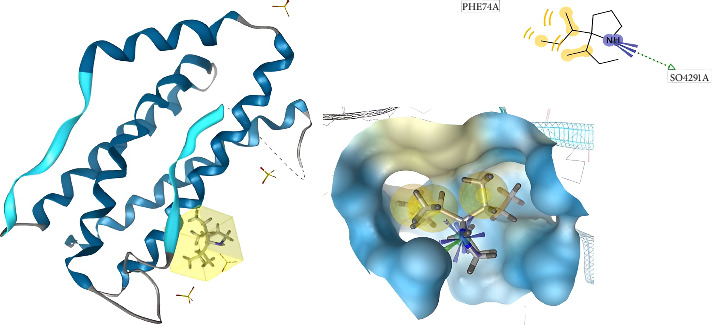
2,3-bis (1-methylallyl) pyrrolidine conformation in the binding site of IL-6 (3D visualization–A and 2D visualization–B).

Figure 9 shows the conformation of 2,3-bis (1-methylallyl) pyrrolidine in the binding site of IL-6. Based on this picture, Phe74 is essential for enhancing ligand affinity. Therefore, it is necessary to enhance the ligand affinity between the native ligand (L (+) -tartaric acid) and the IL-6 receptor binding site.

**Figure 10:**
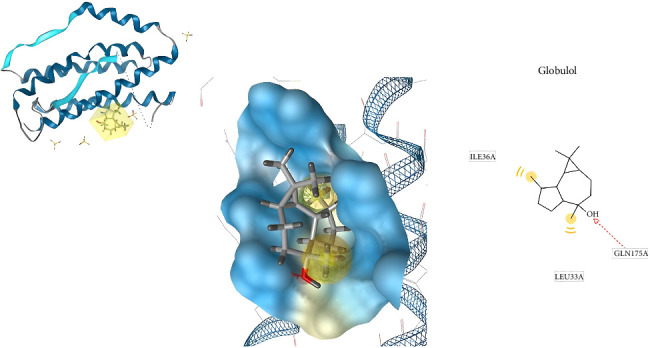
(−)-Globulol conformation in the binding site of IL-6 (3D visualization–A and 2D visualization–B).

Figure 10 shows the conformation of (−) -globulol in the binding site of IL-6. Molecular docking posing for native ligand (L (+) -tartaric acid) with IL-6 receptor binding site shows several important amino acid residues such as Gln175, Arg179, and Arg182. Through comparison with (−) globulol conformation in the binding site of IL-6 (Figure 10), Ile36A, Leu33A, and Gln175 were found to play an important role in enhancing ligand affinity.

### 3.6. Molecular Dynamic Simulation

Molecular dynamic simulation was conducted to explore the atoms' and molecules' movement over a given period. This simulation was performed on pinostrobin chalcone that had the lowest binding affinity energy (−6.08 kcal/mol). The RMSD (root mean square deviation) measured the difference of the protein backbones from initial structural conformation to the final position. The deviations observed showed the protein relative stability to the conformation. The molecular dynamic simulation was conducted over 100 ns, and the result is shown in Figure 10.

**Figure 11:**
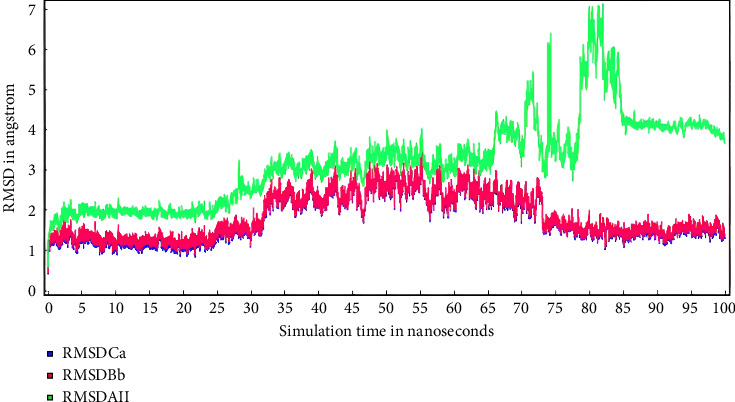
Solute RMSD from the starting structure IL-6 protein.

Figure 11 shows that conformation was stable from 35 to 65 ns followed by a fluctuation in 65 to 85 ns, and stability at > 85 ns. RMSD of Bb and Ca showed only a few fluctuations from 30 to 35 ns, and from 70 to75 ns with RMSD of 0 to 2.5 Å.

**Figure 12:**
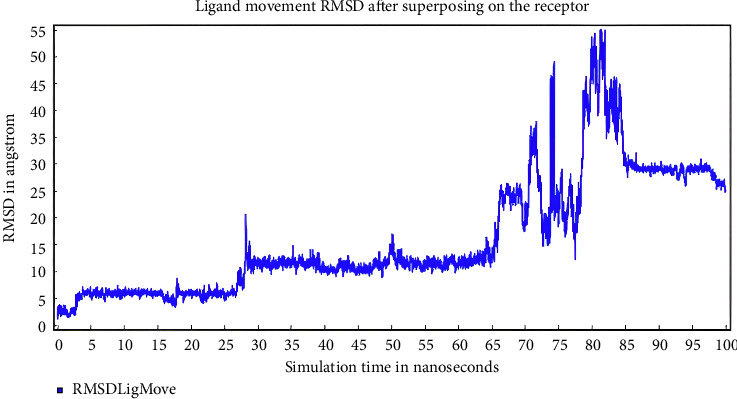
Ligand movement RMSD after superposing on the receptor (pinostrobin chalcone).

Figure 12 shows that RMSD of the ligand was stable at 5–25 ns followed by an increase at 25–30 ns, and stability at 35–65 ns. After 65 ns, RMSD started to increase until 85 ns followed by stability beyond 85 ns.

After 100 ns, the RMSD was evaluated, with IL-6 protein having a range of 0–7.0 Å. This was divided into Ca for C alpha (blue line), Bb for backbone (red line), and RMSD for all (green line) (Figure 11). Based on the ligand movement after superposing, the receptor (IL-6) has a range from 0–55.0 Å (Figure 12).

**Figure 13:**
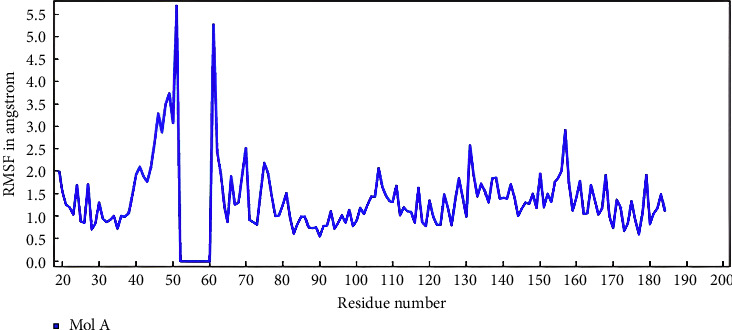
Solute protein/nucleic acid residue RMSF of complex IL-6 and pinostrobin chalcone during 100 ns.

Figure 13 shows the root mean square fluctuation (RMSF) after a 100 ns molecular dynamics simulation, where there were very low fluctuations in the positions of the amino acid residues Gln175, Ile36, Lys171, Asp34, and Arg30. The result implies that the ligand conformation and interaction with the protein were stable at 100,000 ps. The fluctuations in these five amino acids were ≤ 0.5 Å. This picture was the result of a molecular dynamics simulation using YASARA version 22.9.24 (licensed to fadilah.msi@ui.ac.id).

## 4. Discussion

COVID-19 can develop into severe pneumonia with cytokine storm, ARDS, DIC (disseminated intravascular coagulation), and multiorgan failure. Cytokine storm is a severe molecular events' complicated network induced by a lot of white blood cells' activation such as B-cells, monocytes, macrophage, T-cells, dendritic cells, NK-cells, neutrophils, epithelial, and endothelial cells. These events trigger cytokine storm through the immune system hyperactivation and uncontrolled cytokines production, including IFN-*γ*, IL-6, IL-17, IL-12, IL-1*β*, and TNF-*α* [27].

This study identified 30 nodes associated with cytokine storm, ARDS, and inflammatory process of COVID-19, namely, IL-6, IL-6R, NF-*κ*B, IL-2, IL-2RA, IFNA2, IFNAR1, COX5A, and COX411. The nodes are proteins that play a role in cytokine storm and could be a target for interaction with active compounds of *M. cajuputi* leaves ethanol extract. According to several studies, IL-6 takes a part in hyperinflammation and cytokine storm. This study used IL-6 as protein target for interaction with active compounds.

Generally, there are two receptor types for IL-6, namely, membrane-bound IL-6R (mIL-6R) and soluble IL-6R (sIL-6R). There are also three IL-6 signaling types, namely, classical, trans, and transpresentation. IL-6 binds to the mIL-6R and forms the IL-6-mIL-6R complex, which then combines gp130 to produce signal transduction by the JAK-STAT pathway, called as classical signaling. Moreover, IL-6 binds to sIL-6R to produce IL-6-sIL-6 complex and combines to gp130 to generate signal transduction called transsignaling. In general, transsignaling promotes proinflammatory events, while classical signaling triggers anti-inflammatory events [28, 29]. Increased levels of IL-6 in serum can be used as a predictor of COVID-19 progression; hence, blocking this protein is considered an effective treatment to prevent cytokine storm and ARDS [10, 30].

In this study, *M. cajuputi* leaves were extracted, followed by profiling of active compounds. Using GC-MS, eight main active compounds were obtained including pinostrobin chalcone, 1-acetyl-2,2,6,6-tetramethyl-4-acetyloxypiperidyne, hexadecanoic acid, 5H-indeno [1,2-b] pyridine, 4-methyl, 2- (trimethylsilyl) benzenethiol, (−) -Gglubolol, (2,3-bis (1-methylallyl) pyrrolidine), and (1,4-benzenediol,2-methyl-,4-acetate). All compounds were confirmed to be nontoxic and met the Lipinski rule of five for drug-likeness and ADME.

Molecular docking was performed to examine compounds' interactions with IL-6. Among the active compounds, pinostrobin chalcone was found to interact significantly with IL-6. Chalcone or (E)-1,3-diphenyl-2-propene-1-one scaffolds have received great scientific interest in medicinal chemistry due to the chemical simplicity, ease of synthesizing various derivatives, and promising pharmacological activities by modulating multiple molecular targets. Several natural and (semi-) synthetic chalcone derivatives have shown significant anti-inflammatory activity because of the various therapeutic-targets potential inhibition such as cyclooxygenase (COX), lipooxygenase (LOX), IL, prostaglandins (PGs), nitric oxide synthase (NOS), leukotriene D4 (LTD4), nuclear factor-ΚB (NF-ΚB), intracellular cell adhesion molecule-1 (ICAM-1), vascular cell adhesion molecule-1 (VCAM-1), monocyte chemoattractant protein-1 (MCP-1), and TLR4/MD-2. Moreover, chalcone scaffolds with carboxyl, hydroxyl, heterocyclic, prenyl, and methoxyl ring substitutions such as thiophene/furan/indole have shown promising anti-inflammatory activities [31].

This study used dexamethasone as an anti-inflammatory drug standard. Dexamethasone can inhibit the expression of cytokines in COVID-19 patient including IL-6. The level of IL-6 decreased in COVID-19 patient plasma during dexamethasone treatment [32]. In this study, the molecular docking result showed that dexamethasone had an interaction with amino acid in IL-6 binding site. Its binding affinity score is low (−5.27 kcal/mol). Meanwhile, the binding affinity score of pinostrobin chalcone-IL-6 was lower than dexamethasone. Therefore, it is possible that pinostrobin chalcone has a potential anti-inflammatory activity.

Molecular docking and dynamic simulation (100 ns) showed that there was a specific inhibitor interaction between pinostrobin chalcone and IL-6. This interaction suggests the *M. cajuputi*'s ethanol extract has the potential to inhibit IL-6. The binding energy of pinostrobin chalcone was better than that of the native ligand as a positive control. Based on the results, the binding of IL-6 as a ligand with pinostrobin chalcone was stable after 85 ns. The RMSF results showed that the pinostrobin chalcone bound amino acids with a range of ≤ 0.5 Å, indicating stable interactions with Gln175, Ile36, Lys171, Asp34, and Arg30. These amino acids play an important role in enhancing ligand affinity. Based on these results, pinostrobin chalcone from the ethanol extract of *M. cajuputi* leaves has great potential as an anti-inflammatory agent due to the ability to block IL-6 activity.

The binding of IL-6 with pinostrobin chalcone blocks interaction with the sIL-6R and mIL-6R. Consequently, the interaction between IL-6/IL-6R with gp130 does not occur, referring to the classic and transsignaling inhibition. This inhibition blocks the activation of Janus kinase (JAK)-STAT3 along with proinflammation and anti-inflammatory events [33, 34].

Blocking IL-6-IL-6R binding leads to acute phase response inhibition, impeding the *α*-1 antitrypsin (AAT) production and release which has a protective endogenous antiprotease and anti-inflammatory function, increasing the risk of infection [28, 35]. Consequently, considering the possible side effects of blockade in subsequent in vivo or in vitro studies is essential.

This study only used the normal IL-6 structure for molecular docking and dynamic simulation. However, mutation in the gene, specifically in the amino acids representing the active site, could affect interaction with pinostrobin chalcone. The IL-6 gene polymorphism could also change the binding of the pattern [36]. In this study, gene polymorphism was not included in molecular docking and dynamic analysis. Therefore, further studies should include the gene polymorphism structure and the gene mutation structure for analysis.

## 5. Conclusion

In conclusion, *M. cajuputi* showed potential anti-inflammatory activity against COVID-19 by blocking IL-6 activity. IL-6 is protein that plays a role in the inflammatory process and cytokine storm; hence, blocking the activity can prevent complications. Based on the results, active compounds of *M. cajuputi* interacting with IL-6 include pinostrobin chalcone, 1-acetyl-2,2,6,6-tetramethyl-4-acetyloxypiperidyne, hexadecanoic acid, 5H-indeno [1,2-b] pyridine, 4-methyl, and 2-(trimethylsilyl) benzenethiol. Among these compounds, pinostrobin chalcone had the best interaction with IL-6, as shown by the lowest delta G value.

## Figures and Tables

**Table 1:** Chemical composition of liquid extract of *M. cajuputi* leaves from GC/MS.

Retention time	Quality	Compounds	PubChem ID	Composition (%)
6.797	96	(+)-Dipentene	440917	2.77
10.403	93	2,3-dihydro-3,5-dihydroxy-6-methyl-4H-pyran-4-one	119838	2.79
27.531	46	4-(ethoxymethyl) -2-methoxyphenol	61586	3.36
28.379	30	2-chloro-5-methoxybenzimidazole	519197	3.26
28.620	72	2-(Trimethylsilyl) benzenethiol	N/A	5.49
29.372	38	1-Acetyl-2,2,6,6-tetramethyl-4-acetyloxypiperidyne	579916	33.32
29.370	58	Methyl beta-d-galactopyranoside	94214	41.82
30.082	91	Hexadecanoic acid	985	3.48
30.675	45	5H-indeno [1,2-b] pyridine, 4-methyl	183441	1.51
33.178	96	Pinostrobin chalcone	5316793	1.39
12.57	N/A	Eucalyptol	2758	8.64
24.31	N/A	Caryophyllene	5281515	2.80
28.41	N/A	((−)-Globulol)	12304985	9.67
36.30	N/A	n-Hexadecanoic acid	N/A	6.48
33.52	N/A	Cryptomeridiol	165258	5.16
32.76	N/A	2,3-bis (1-methylallyl) pyrrolidine	5205941	6.91
27.79	N/A	Beta-D-glucopyranoside, methyl	36462	12.81
32.76	N/A	(1,4-benzenediol,2-methyl-,4-acetate)	15532447	8.21

**Table 2:** Hub genes analysis.

ENSP ID	Gene symbol	MCC	MNC	Betweenness
ENSP00000226574	NFKB1	4843440	22	48,038
ENSP00000385675	IL6	4839156	25	113,714
ENSP00000269305	TP53	4829072	21	101,444
ENSP00000354394	STAT1	4829040	21	42,753
ENSP00000437955	HIF1A	4803150	17	45,333
ENSP00000384273	RELA	4790166	19	30,598
ENSP00000495360	CTNNB1	4768608	18	35,418

**Table 3:** Molecular docking results.

Compounds	Binding affinity (kcal/mol)	Ki (uM)
L (+)-tartaric acid (native ligand)	−5.40	110.71
(+)-dipentene	−4.30	705.66
2,3-dihydro-3,5-dihydroxy-6-methyl-4H-pyran-4-one	−4.50	501.68
4-(ethoxymethyl)-2-methoxyphenol	−4.25	766.37
2-chloro-5-methoxybenzimidazole	−4.46	535.76
2-(Trimethylsilyl) benzenethiol	−4.88	265.1
1-Acetyl-2,2,6,6-tetramethyl-4-acetyloxypiperidyne	−5.21	266.58
Methyl beta-d-galactopyranoside	−3.13	5040
Hexadecanoic acid	−5.08	188.32
5H-indeno [1,2-b] pyridine, 4-methyl	−5.07	193.58
Pinostrobin chalcone	**−6.08**	**34.77**
(−)-Glubolol	−5.00	217.39
(2,3-bis (1-methylallyl) pyrrolidine)	−5.90	47.22
(1,4-benzenediol,2-methyl-,4-acetate)	−5.57	82.94

*Note:* The bold of number -6.08 means it is the lowest binding affinity. It is the best interaction between IL-6 and active compounds. Ki is the inhibition constant. It is a value that represents the dissociation constant of an enzyme inhibitor complex that has been docked. A smaller Ki value indicates a lower probability of dissociation, which means higher inhibition. Therefore, 34,77 is the best of Ki. Lower KI values imply stronger binding affinity, meaning the ligand binds more effectively to the target. Higher KI values indicate weaker binding, suggesting less effective interaction.

## Data Availability

Data supporting the results are included within the article.

## Supporting Information

Table S1 contains drug-likeness and ADMETox prediction using SwissADME and ProTox II. All active compounds are mostly nontoxic, with toxicity classes between 4 and 5. In addition, the compounds meet the drug-likeness criteria (hydrogen bond donor, hydrogen bond acceptor, logP, and molecular weight) and are noninhibitors for CYP P450.
