# Supplementary material for: Protein Interaction Analysis and Molecular Simulation of the Anti-Inflammatory Activities in Melaleuca cajuputi Extract Against COVID‐19
Source: Int J Inflam. 2024 Nov 28;2024:5568294. doi: 10.1155/ijin/5568294 (PMC11620808; doi:10.1155/ijin/5568294)
Supplement: Supporting Information — Additional supporting information can be found online in the Supporting Information section. [file 5568294.f1.pdf]

SUPPLEMENTARY FILE

Table S1. Drug-likeness and ADMETOX prediction

Compounds	Drug-likeness				Toxicity class	Toxicity			Pharmacokinetics				
	MW	HBA	HBD	Log P		Hepatotoxicity	Carcinogenicity	Mutagenicity	CYP 1A2	CYP2 C19	CYP 2C9	CYP 2D6	CYP 3A4
(+)-Dipentene	136.23	0	0	3.31	5	no	no	no	no	no	no	no	no
2,3-dihydro-3,5-dihydroxy-6-methyl-4H-pyran-4-one	144.13	4	2	-0.26	4	no	no	yes	no	no	no	no	no
4-(ethoxymethyl)-2-methoxyphenol	182.22	3	1	1.66	5	no	no	no	no	no	no	no	no
2-chloro-5-methoxybenzimidazole	182.61	2	1	1.95	3	yes	no	no	yes	no	no	no	no
2-(trimethylsilyl) benzenethiol	617.2	1	0	6.93	5	no	no	no	no	no	no	no	no
1-acetyl-2,2,6,6-tetramethyl-4-acetyloxypiperidyne	241.33	3	0	1.86	5	no	no	no	no	no	no	no	no
Methyl beta-d-galactopyranoside	194.18	6	4	-1.64	6	no	no	no	no	no	no	no	no
Hexadecanoic acid	256.42	2	1	5.20	4	no	no	no	yes	no	yes	no	no

5H-indeno [1,2-b] pyridine, 4-methyl	270.33	2	0	4.04	4	no	no	yes	yes	yes	no	no	yes
Pinostrobin chalcone	270.28	12	2	3	4	no	no	no	yes	no	yes	no	yes

The table above shows that all of active compounds are mostly nontoxic whereas the toxicity class is between 4-5, and the compounds meet the drug-likeness criteria (hydrogen bond donor, hydrogen bond acceptor, logP, and molecular weight) and non-inhibitor for cyp P450
